# Continuing Trend of Increased Operative Treatment of Clavicle Fractures in Pediatric Patients in the United States Despite a Lack of Supporting Evidence in the Literature

**DOI:** 10.3390/children13081075

**Published:** 2026-08-14

**Authors:** Mahara Mohseni, Jad Lawand, Alireza Mirahmadi, Rohit Siddabattula, David Momtaz, Kaveh Momenzadeh, Abdullah Ghali, Mohammad Javad Shariyate, Pooya Hosseinzadeh

**Affiliations:** 1 Department of Orthopedic Surgery, School of Medicine, Washington University, St. Louis Children’s Hospital, St. Louis, MO 63110, USA; mahara@wustl.edu (M.M.);; 2 John Sealy School of Medicine, The University of Texas Medical Branch, 301 University Blvd, Galveston, TX 77555, USA; jjlawand@utmb.edu (J.L.); rs8dy@umsystem.edu (R.S.); 3 Musculoskeletal Translational Innovation Initiative, Carl J. Shapiro Department of Orthopaedic Surgery, Beth Israel Deaconess Medical Center and Harvard Medical School, Boston, MA 02215, USA; amirahma@bidmc.harvard.edu (A.M.);; 4 Department of Orthopaedic Surgery, University of Miami, Miami, FL 33146, USA; 5 Department of Orthopaedic Surgery, Baylor College of Medicine, Houston, TX 77030, USA

**Keywords:** clavicle, pediatric, adolescent, fracture

## Abstract

**Highlights:**

**What are the main findings?**

- ORIF utilization for pediatric and adolescent clavicle fractures increased from 1.62% in 2014 to 5.89% in 2024, representing a 263.6% increase.
- The rise was concentrated among patients aged 15–18 years, whose ORIF rate increased from 3.4% to 12.4%, while rates among patients aged 10–14 years remained relatively stable.
- Male and White patients had higher overall ORIF rates, although operative utilization increased more rapidly among female and Black patients, narrowing some demographic differences over time.

**What are the implications of the main findings?**

- Operative treatment is increasingly used despite limited evidence that routine fixation provides superior long-term outcomes over nonoperative management in pediatric and adolescent patients.
- The findings support more selective surgical indications, careful shared decision-making, and consideration of implant-related complications, secondary procedures, and healthcare costs.
- Demographic variation in ORIF utilization suggests that factors beyond fracture characteristics may influence treatment decisions, highlighting the need for evidence-based and equitable treatment guidelines.

**Abstract:**

**Introduction**: Pediatric clavicle fractures are among the most common fractures in this age group. Current research supports the efficacy of nonoperative treatment for these injuries in pediatric patients. Our study seeks to examine the current trends in the management of these fractures and determine whether they align with the existing evidence-based guidelines. **Methods**: A retrospective analysis of pediatric patients with clavicle fractures between 1 January 2014 and 31 December 2024 was analyzed. A retrospective query was performed using the TriNetX Research Network. Patients were dichotomized into two groups based on open reduction internal fixation and nonoperative or closed treatment codes over this period, performing subgroup analyses by sex, race, and age. **Results**: We evaluated 78,251 patients between 10 and 18 years with clavicle fractures from 2014 to 2024, of whom 3546 underwent open reduction and internal fixation (ORIF). ORIF utilization increased from 1.62% in 2014 to 5.89% in 2024, representing a 263.6% increase from the 2014 baseline. Males consistently underwent ORIF more often than females. Adolescents (15–18 years) had substantially higher and faster-rising ORIF rates than younger patients (10–14 years), increasing from 3.4% to 12.4% versus 0.8% to 2.5% between 2014 and 2024 (APC 7.5% vs. 0%, *p* = 0.001). White patients had higher ORIF rates than African American patients, although some disparities narrowed over time. **Conclusions**: Despite limited supporting evidence, current trends demonstrate a substantial increase in the surgical management of pediatric clavicle fractures, with operative treatment rising by 263.6% by 2024. This increase in ORIF procedures may expose patients to potentially avoidable risks, including infection, anesthesia-related complications, implant-related issues, and prolonged recovery, without clearly established benefits in many cases.

## 1. Introduction

The clavicle is one of the most commonly fractured bones in children and adolescents, occurring at a rate of 29.14 per 100,000 individuals annually and accounting for 2.6–4% and 8–15% of all fractures in adults and adolescents, respectively [1,2]. The age group of 13 to 20 years demonstrates a notably higher incidence, likely due to their higher participation in sports and other high-risk activities [1,3]. The leading causes are contact injuries or direct trauma (60%) during sports activities (66%), with football, rugby, and soccer being the most commonly associated sports [3,4].

In skeletally immature patients, most clavicle fractures may be efficiently managed through non-operative means, particularly those displaying minimal displacement, which has been shown to have a low rate of nonunion [5]. The management strategy for adolescent diaphyseal clavicle fractures has experienced significant transformations in recent decades, displaying a clear tendency towards surgical procedures [6,7,8,9,10,11,12]. Between 1980 and 2000, Kubiak et al. reported 1.6% surgical rate in children and adolescent (1–16 years old) clavicular fracture [13]. However, later in 2020, Ellis et al. by a prospective multicenter study in collaboration with the FACTS study group reported that 32% of displaced clavicles and 17% of all clavicular fractures in adolescent (age 10–18), irrespective of displacement, were subjected to surgical management [3]. These changing patterns closely resemble the developing approaches in managing clavicle fractures in adults, which have been shaped by randomized controlled trials primarily involving adult participants. However, the absence of published randomized controlled trials directly comparing surgical and non-surgical methods in adolescents is worth noting.

The growing preference for surgical interventions may not align with the well-documented exceptional healing capability of the clavicle and goes against the most recent prospective investigations on clavicle fractures in children and adolescents [5,14,15,16,17,18]. Therefore, there has been a dramatic increase in operative treatment over the past years, but no consensus guidelines exist for operative versus non-operative management of these injuries [19,20].

The primary objective of this study was to better characterize the epidemiology of clavicle fractures in pediatric and adolescent populations using data from the TriNetX Research Network, a large multicenter database.

Specifically, our objectives were to investigate the distribution of fractures and the demographic characteristics of the affected individuals, thereby providing updated insights based on the most recent data available. Furthermore, given the trend towards surgical interventions in contrast to existing literature recommendations, this study aimed to assess any potential shifts in the rates of surgical versus non-surgical management during this period. Our hypothesis was that the utilization of surgical treatments in clavicle fractures has increased over time.

## 2. Materials and Methods

We utilized TriNetX Research Network, a comprehensive database of deidentified electronic medical records aggregated from 64 healthcare organizations in the US Collaborative network. TriNetX is a federated research network containing de-identified electronic health record data contributed by participating healthcare organizations. Patients are represented in the network through clinical encounters at participating institutions rather than through direct research recruitment. The network includes data from multiple healthcare settings; however, participating organizations are not selected through national probability sampling. The cohort was not restricted to hospitalized patients. TriNetX includes inpatient and outpatient electronic health record data, including ambulatory clinic, emergency department, inpatient, observation, and other documented encounters. The database was queried in January 2025. Our study focused on patients aged 10 to 18 who sustained clavicle fractures between 1 January 2014, and 31 December 2024. Patients with clavicle fractures were identified using ICD-9-CM code 810 and its subcodes for encounters before 1 October 2015, and ICD-10-CM code S42.0 and its subcodes for encounters on or after 1 October 2015. We analyzed treatment modalities, specifically open reduction internal fixation (ORIF) with CPT code 23515, and closed treatments with CPT codes 23500 and 23505.

Our analysis encompassed a comparative evaluation of the incidence and changes in treatment modalities for surgical and non-surgical treatment from 2014 to 2024. Subgroup analyses were conducted by sex, race, and age. Regression modeling was used to evaluate temporal changes in treatment utilization across the study period.

Given that only deidentified patient records were used and there was no access to personally identifiable information, this study was exempt from Institutional Review Board (IRB) approval.

The percentages of surgical procedures were calculated annually for the total patient population and then for each demographic category, including age (10–14, 15–18), sex (female, male) and race (African American, White). Chi-squared analysis was conducted to investigate the associations between demographic categories and the type of surgical intervention (surgical vs. non-surgical) from 2014 to 2024. Thereafter, temporal trend analysis of the surgically treated proportion of the total patient population from 2014 to 2024 was conducted using Joinpoint regression analysis. This method segmented the time series data into distinct intervals where the rate of increase or decrease could differ. Within each interval, a separate linear regression model described the surgically treated proportion percentage. The analysis determined the Annual Percent Change (APC), enabling the detection of significant changes in the trend within the predefined time points.

Once the Join point year was identified, the corresponding year of trend change was established as the baseline for subsequent comparative analyses of surgical trends across subgroups.

Subsequent annual percentages were then compared to the baseline year to determine the percentage change over time. A linear regression analysis was performed on the percentage change from the baseline, using the year as the independent variable. The regression coefficients suggest a per-year change in surgical percentages, with a constant term representing the estimated percentage at the baseline year. To extend the analysis beyond the available data, the observation period was expanded through 2030, and projected values for future years were estimated using the established regression model.

The Joinpoint analysis was conducted in the Joinpoint regression software (Version 5, Statistical Research and Applications Branch, National Cancer Institute, Bethesda, MD, USA). All other statistical analyses were performed utilizing Stata software (Version 18.0, StataCorp LP, College Station, TX, USA) and R (version 4.4.2, Foundation for Statistical Computing, Vienna, Austria) package. To account for multiple annual comparisons, a Bonferroni correction was applied separately to the 11 Chi-square tests conducted for each demographic category. Accordingly, statistical significance for these analyses was defined as *p* < 0.0045.

## 3. Results

The TriNetX query of patients between 10 and 18 who sustained clavicle fractures between 2014 and 2024 identified 78,251 patients, of whom 3546 underwent an ORIF procedure.

### 3.1. Total Population

In the descriptive analysis of surgical procedures from 1 January 2014, and 31 December 2024, we observed varying percentages across patient demographics (Table 1).

Annual chi-squared (χ^2^) tests were performed to assess yearly associations between demographic characteristics and surgical intervention type from 2014 to 2024. Comparisons were conducted between African American or Black and White patients, male and female patients, and two age groups: 10–14 years and 15–18 years. A significant association between age group and surgical intervention type was observed in every year analyzed (all *p* < 0.001). Race was significantly associated with intervention type in most years, except 2014 and 2024. Sex showed a less consistent association, reaching statistical significance in 2014–2016, 2020, and 2021, but not in 2017–2019 or 2022–2024 (Table 2).

**Figure 1:**
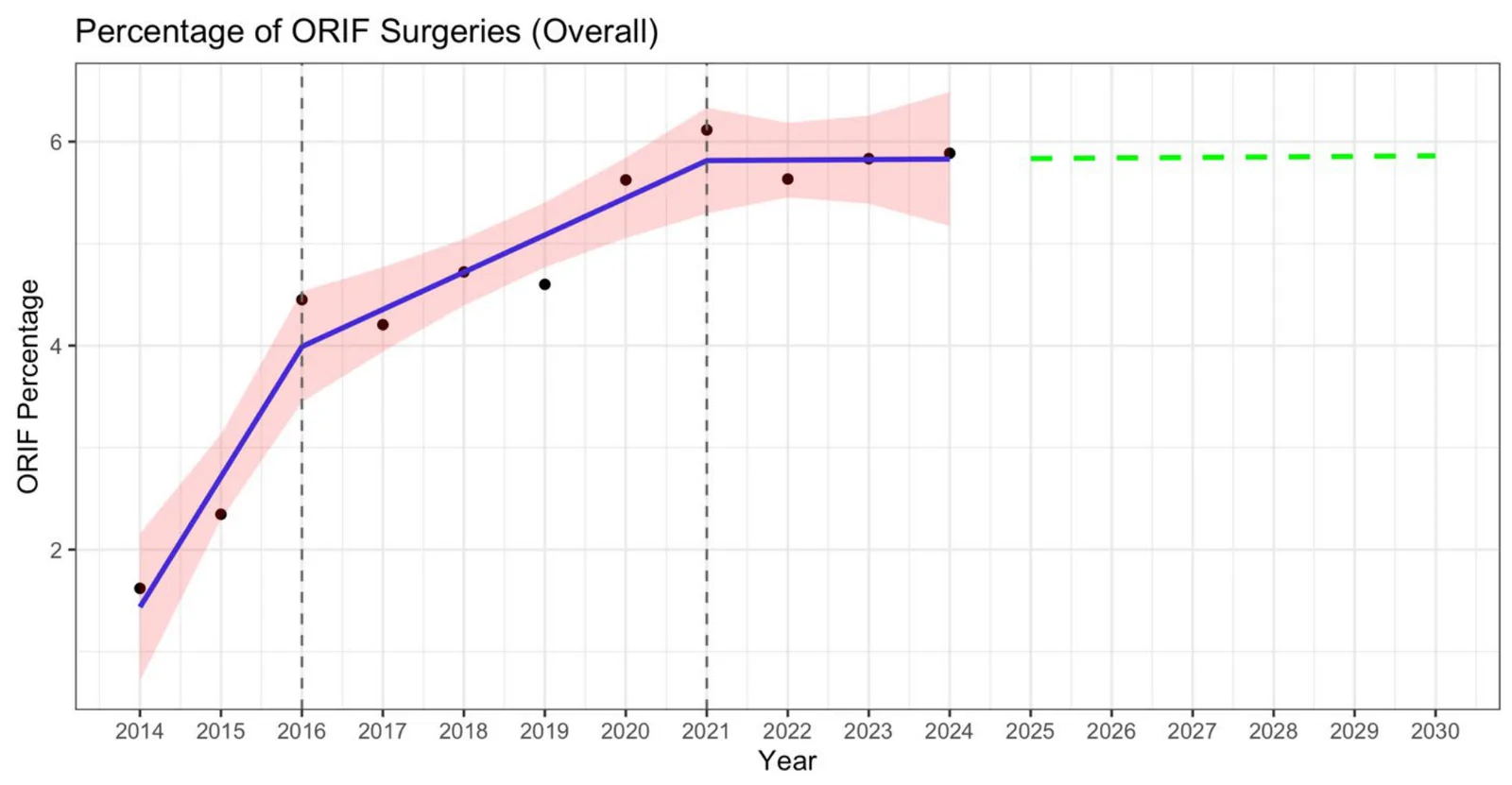
Joinpoint regression analysis of the overall percentage of open reduction and internal fixation (ORIF) surgeries among patients with clavicle fractures. Black dots represent the observed annual ORIF percentages, and the solid blue line represents the fitted Joinpoint regression trend. The red shaded area indicates the 95% confidence interval around the fitted trend. Vertical gray dashed lines indicate the identified joinpoints (2016 and 2021), where a significant change in the temporal trend was detected. The green dashed line represents the projected ORIF percentage for 2025–2030 based on continuation of the final fitted trend segment.

Overall, the percentage of all patients undergoing surgical treatment showed a gradual increase from 1.62% in 2014 to 5.89% in 2024. In the Joinpoint regression analysis for the overall population, a notable shift in the trend of surgical procedures was identified in the year 2016, marking it as a pivotal year where the trend in surgical treatment incidence rates significantly changed (Figure 1).

A consistent increase in the frequency of surgeries was observed from 2014 to 2016 with Annual Percentage Change (APC) of 61.27%. However, between 2016 and 2021, the trend showed a smaller increase in the annual change in ORIF surgeries with APC of 7.39%. A second trend shift was detected in 2021, after which the annual percentage change decreased to 0.11% after 2021. A regression analysis of the overall data using a piecewise linear model with trend shifts in 2016 and 2021 showed that the surgical percentage increased by 46.1% from 2016 to 2024.

Using 2016 as the baseline, regression analyses were conducted to examine the annual percentage change in surgical treatment across various demographic categories. The percentage change was calculated by comparing each year’s surgical percentage relative to that of the baseline year. Notably, each category demonstrated unique trends over time (Table 3).

### 3.2. Age

#### Category

**Figure 2:**
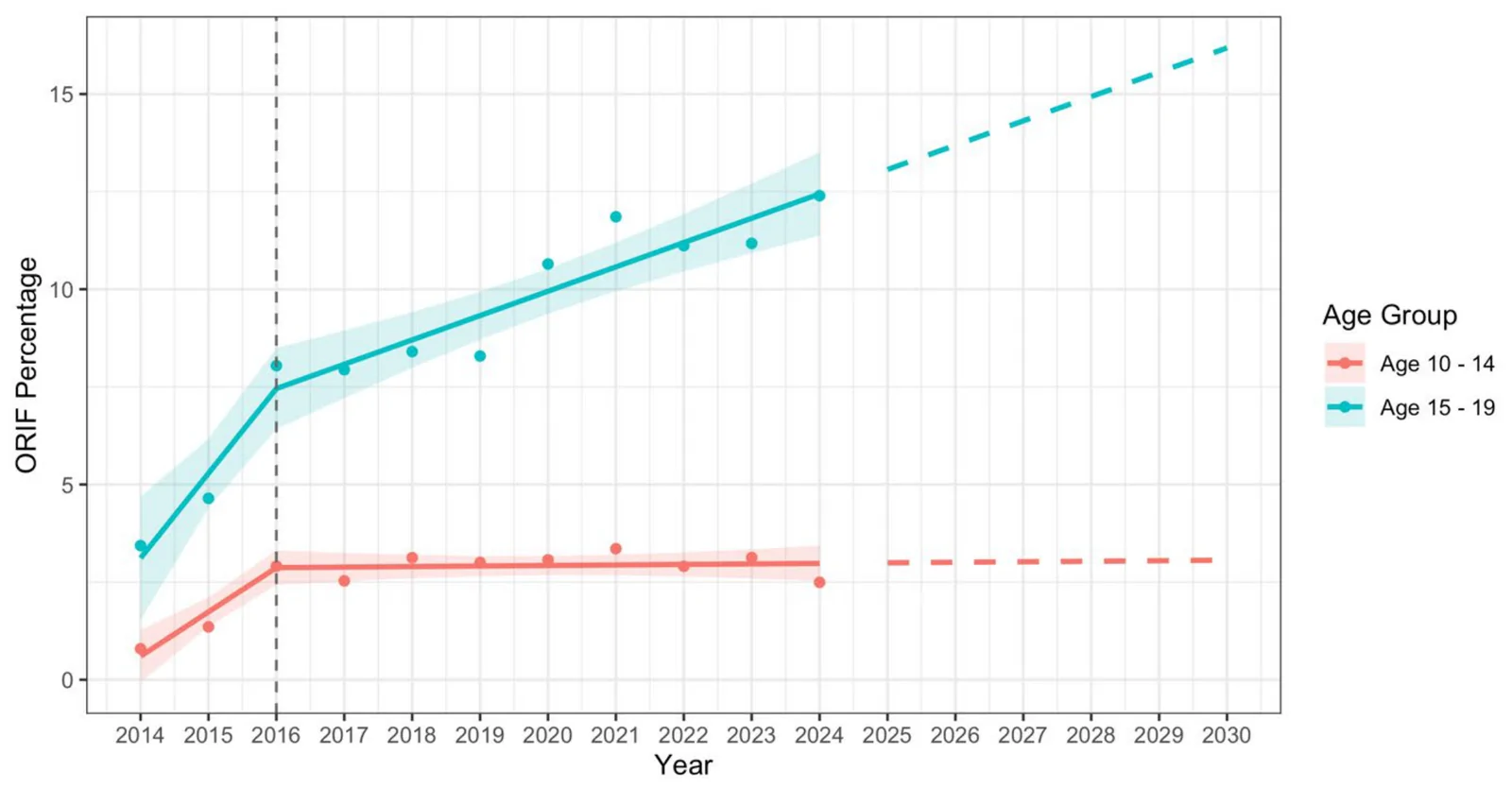
Trends in annual open reduction and internal fixation (ORIF) utilization by age group. The points represent the observed annual ORIF percentages, while the solid lines represent the fitted trends during the observed study period (2014–2024). The shaded areas indicate the corresponding 95% confidence intervals. The vertical gray dashed line indicates the identified joinpoint in 2016. The colored dashed lines extending from 2025 to 2030 represent the projected ORIF utilization based on extrapolation of the final fitted trend for each age group. ORIF utilization remained relatively stable among patients aged 10–14 years but increased substantially among older adolescent patients.

Age-specific surgical percentages indicated that patients aged 15–18 consistently had a higher percentage of surgical procedures compared to those aged 10–14, with the gap widening over time. In 2014, the percentages were 0.8% for the 10–14 age group and 3.4% for the 15–18 age group. This difference peaked in 2024 with 2.5% for the 10–14 age group and 12.4% for the 15–18 age group, suggesting a higher propensity for surgical treatment in the older age range (Figure 2).

Age groups Chi-square comparisons between 10 and 14 years old and 15–18 years old demonstrated a strong and consistent association throughout the years from 2014 to 2024, indicating a very strong link between the age group and the type of surgical intervention (*p* < 0.05).

The 15–18-year-old group showed a significant annual increase in surgical treatment, with an APC of 7.5% (R^2^ = 0.84, *p* < 0.01). In contrast, the 10–14-year-old group showed no significant change over time, with an APC of 0% (R^2^ = 0, *p* > 0.05), indicating a stable rate of surgical treatment during the study period. In the comparison of surgical percentage change trends between the two age groups, there was a statistically significant difference in the annual percentage change in surgical treatment between age groups (*p* = 0.001), suggesting divergent trends in surgical procedure rates over time for these age groups.

### 3.3. Sex Category

**Figure 3:**
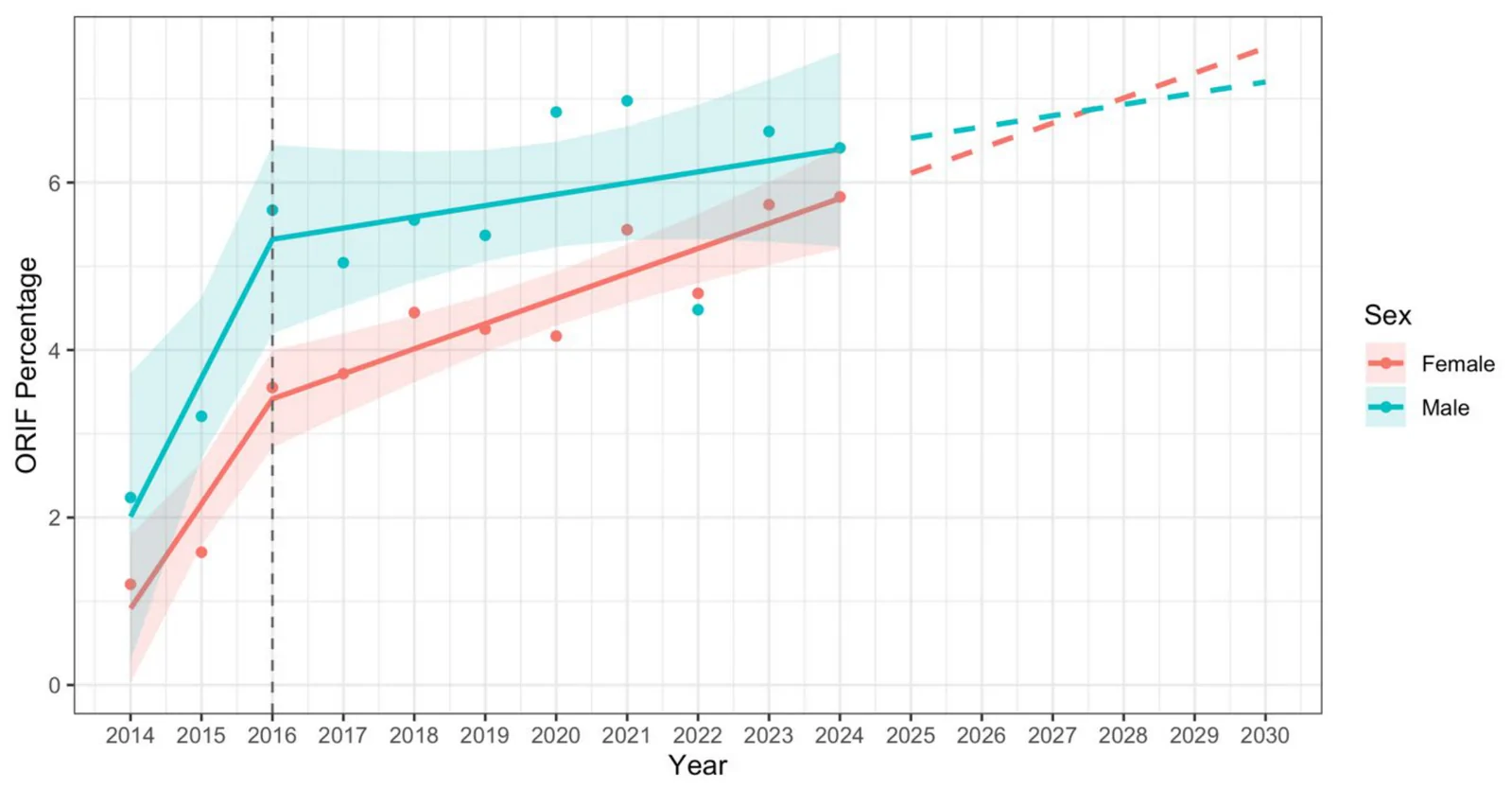
Trends in ORIF Surgery Percentage Changes by Sex Category. The blue solid line represents the observed annual ORIF percentage among female patients, and the red solid line represents male patients. The corresponding dashed lines represent the fitted linear trends. The colored dashed lines extending from 2025 to 2030 represent the projected ORIF utilization based on extrapolation of the final fitted trend for each sex group. Male patients generally had higher ORIF utilization, although the rate of increase over time was steeper among female patients.

Regarding sex, females had a lower percentage of ORIF procedures compared to males across the study period. While the percentage for females increased from 1.2% in 2014 to 5.8% in 2024, the percentage for males increased from 2.2% in 2014 to 6.4% in 2024 (Figure 3).

Interestingly, Chi-square comparisons by sex comparisons indicated a significant association between being male or female and the type of surgical intervention administered in the earlier years, specifically from 2014 to 2016; however, this association diminished starting in 2017 and then in more recent years from 2022 to 2024. Notably, although at a lower rate of ORIF relative to males, females showed a more significant APC with a regression coefficient of 7.9% (R^2^ = 0.82, *p* < 0.01), while males had a coefficient of 2.1% (R^2^ = 0.14, *p* > 0.05), both indicating yearly increases from the baseline but with females experiencing statistically significant and a steeper incline. As such, in the comparison of ORIF percentage change trends between the two sex groups, the analysis indicates a statistically significant difference in the slopes of their trend lines ((F (1, 14) = 5.89, *p* = 0.029).

There was a 70% increase in ORIF percentage among female patients in 2024 relative to 2016, while the increase was only 20% for the male patient population.

### 3.4. Racial Category

**Figure 4:**
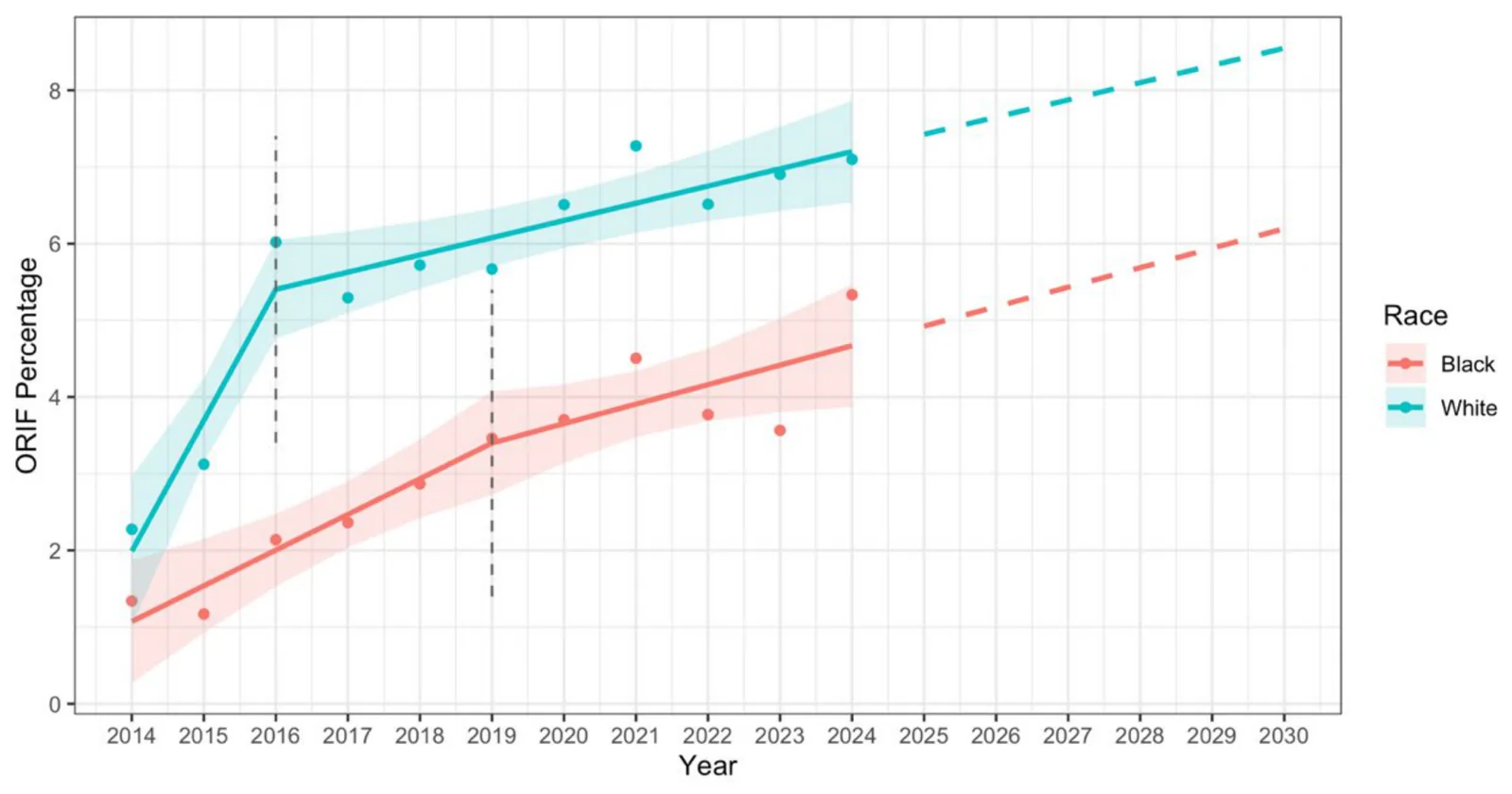
Trends in ORIF Surgery Percentage Changes by Race Category. The blue solid line represents the observed annual ORIF percentage among Black or African American patients, and the red solid line represents White patients. The colored dashed lines extending from 2025 to 2030 represent the projected ORIF utilization based on extrapolation of the final fitted trend for each races. White patients generally had higher ORIF utilization, although the increase relative to baseline was steeper among Black or African American patients.

Racial analysis revealed that Black or African American patients had the lowest percentage of ORIF procedures in 2014 at 1.3%, with a gradual increase to 5.3% in 2024. White patients started at a higher percentage of 2.3% in 2014 and saw an increase to 7.1% in 2024 (Figure 4).

The trend indicates a consistently higher percentage of ORIF procedures among White patients compared to Black or African American patients over the years. However, when looking at the increasing trend of ORIF (APC of 15.0% in Black or African American vs. 3.4% for White category), the analysis indicates that the ORIF percentage change between White individuals and Black or African American individuals is significantly different (F (1, 14) = 12.68, *p* = 0.003). In 2024, relative to 2016, the ORIF percentage increased by 125% for the Black or African American patient population, while this change was 33% for White patients.

## 4. Discussion

We conducted a retrospective analysis of over 78,000 patients aged 10 to 18 years who sustained clavicle fractures between 2014 and 2024. During this period, the rate of operative intervention increased by 263.6%, rising from 1.62% in 2014 to 5.89% in 2024. While previous studies have reported similar upward trends in surgical management despite limited supporting evidence [3], our study is the first to comprehensively evaluate these trends with a focus on racial and gender disparities in addition to the overall increase in surgical treatment.

With respect to sex-based differences, female patients consistently underwent fewer open reduction and internal fixation (ORIF) procedures compared to their male counterparts throughout the study period. Nevertheless, the proportion of females receiving ORIF increased markedly from 1.2% in 2014 to 5.8% in 2024, while the rate among males rose from 2.2% to 6.4% over the same interval. Both sexes exhibited progressive increases in operative intervention relative to their respective baselines; however, the rate of increase among females was significantly steeper and reached statistical significance. While some prior studies have reported no significant differences in surgical rates based on sex, others have observed a higher frequency of operative management in male patients [12,21,22].

Our analysis demonstrated that White patients consistently underwent ORIF at a higher proportion than African American patients throughout the study period (*p* < 0.05). Nevertheless, this racial disparity appeared to diminish over time, as African American patients exhibited a significantly greater annual percent increase in ORIF utilization compared with White patients (APC, 8.8% vs. 3.9%; *p* = 0.002).

In our study, older age (15–18 years), male sex and White race (compared to African American) were associated with a higher likelihood of receiving operative treatment. Zhang et al. [23] reported that approximately 50% of patients aged 15–18 years underwent surgical management for isolated, displaced midshaft clavicle fractures. Their findings indicated that both older age and higher body mass index (BMI) were significant predictors of surgical intervention. Moreover, they noted that patients treated at adult hospitals had over five times the odds of undergoing surgical treatment compared to those managed at pediatric hospitals. Similarly, Luo et al. [24], in a retrospective review of 153 adolescent clavicle fractures in patients aged 14 to 17 years, found that orthopedic trauma surgeons performed surgical fixation at more than three times the rate of their pediatric fellowship-trained counterparts within the same institution.

Cole et al. found that adolescent patients with private insurance were significantly more likely to receive surgical treatment than those with Medicaid [12]. The cause of these differences is outside the scope of this paper; however, this finding may help to explain our observed difference in clavicle treatment by race, with white patients receiving operative intervention more often than African American patients.

Non-displaced clavicle fractures in both adults and children are generally treated nonoperatively, with surgery reserved for absolute indications such as open fractures, threatened skin integrity, or major neurovascular injury. The main area of controversy involves displaced midshaft fractures. Although operative fixation in adults has been supported by concerns regarding nonunion and potential benefits in short-term functional recovery and earlier return to work, these factors are less applicable to pediatric and adolescent patients, in whom clavicle nonunion is exceedingly rare. A 2018 systematic review identified only 21 reported cases of clavicle fracture nonunion among patients aged 4 to 18 years [25]. Therefore, Although the exact denominator is unclear in pediatric and adolescent, the adult rationale for increasing operative fixation based on nonunion risk should not be directly extrapolated to younger patients.

Fracture shortening is often considered an indication for surgical intervention in adult clavicle fractures. However, recent studies involving serial imaging up to the age of 25 years have demonstrated continued clavicular growth in adolescents. In males, the clavicle increases in length by an additional 34% after the age of 12 years, with an average annual growth of 3.2 mm between the ages of 16 and 19. In females, the corresponding values are slightly lower, at 26% and 2.2 mm per year, respectively [26]. These findings underscore the presence of residual growth potential in the adolescent clavicle, suggesting that this population may better tolerate fracture shortening and exhibit meaningful remodeling following injury.

Studies over the past decade have consistently demonstrated that adolescent patients with closed clavicle fractures treated non-operatively achieve outcomes that are equal to or better than those treated surgically, even in cases involving markedly displaced or translated fracture patterns [17,21,27,28]. The FACTS study showed worse outcomes, lower satisfaction and QuickDash scores in surgical group, although the results were not statistically significant [17]. A recent study by Ahearn et al., showed returned to sporting activities for adolescents following non-operative management averaged 61 days (SD 38 days) which aligns with the current recommendation of return to sport between 8 and 12 weeks [29]. Adolescent patients even retain good functional outcomes, despite significant shortening, with Schulz et al. showing that at two years of follow up, compared to the uninjured side, patients had no differences in strength or range of motion [21].

In a study by Li et al., complications following operative management of displaced midshaft clavicle fractures in adolescent patients were reported at an alarmingly high rate. When implant removal was classified as a complication, the overall complication rate reached 86% (32 out of 37 cases), with 59% of complications attributed to plate prominence or irritation. These findings underscore the potential morbidity associated with surgical fixation in this population and highlight the importance of weighing the risks and benefits of operative intervention [30]. Additionally, although rare, refracture after plate removal has been documented in the literature to be as high as 5% [30].

The observed rise in ORIF utilization may subject children and adolescents to unnecessary risks, including infection, anesthesia-related morbidity, prolonged recovery, and frequent secondary procedures for implant removal, without proven long-term functional advantage. Moreover, this trend carries important economic implications, increasing financial burden on families and adding to healthcare system costs. Although direct cost-effectiveness analyses are lacking, the currently available evidence supports the inference that nonoperative management of most pediatric clavicle fractures is likely the more economically favorable approach. In addition to these risks, potentially unnecessary surgical treatment may result in permanent scarring, hospitalization, disruption of school and family activities, and psychological stress for pediatric patients and their caregivers. These physical, emotional, and social burdens should be carefully considered when the benefits of operative fixation over nonoperative management remain uncertain. The database did not provide information regarding the specialty or subspecialty of the treating surgeon. Consequently, we could not determine whether operative fixation was performed by pediatric orthopedic surgeons, adult orthopedic trauma surgeons, or other orthopedic specialists. Differences in exposure to pediatric-specific evidence and treatment recommendations may potentially contribute to variation in operative decision-making; however, this hypothesis could not be evaluated using the available data.

The primary limitations of this study relate to its retrospective nature and reliance on a large administrative database. Detailed clinical variables were not available, including the specific indications for operative intervention, differentiation of isolated fractures from polytrauma cases, insurance coverage, and radiographic features of the fracture like displacement, shortening, comminution and clinical features like open injury, neurovascular compromise, polytrauma, BMI, insurance status, and the documented indication for surgery. Therefore, our findings describe a population-level change in treatment patterns that should be interpreted cautiously in the absence of patient-level clinical and radiographic information. Furthermore, the use of ICD and CPT coding carries an inherent risk of miscoding and misclassification. Collectively, these limitations constrain the clinical specificity of our findings and may affect their interpretation. Prospective investigations with richer patient-level and radiographic data are needed to confirm these results and better define the factors influencing treatment decisions. Because TriNetX includes patients receiving care within participating healthcare organizations, the study population may be affected by selection related to institutional participation, geographic distribution, referral patterns, access to care, coding practices, and data completeness. In addition, payer information was not consistently available; therefore, the representation of patients with commercial insurance, Medicare, Medicaid, or self-pay status could not be assessed. These factors may limit the generalizability of our findings to the overall pediatric population in the United States.

## 5. Conclusions

This study demonstrates a continued increase in operative treatment of pediatric and adolescent clavicle fractures, particularly among older adolescents, despite limited evidence supporting the routine superiority of surgical fixation over nonoperative management in this population. Sex and race were significantly associated with the likelihood of operative treatment, suggesting that factors beyond fracture characteristics may influence treatment decisions. Although surgery remains appropriate for select indications, the increasing use of ORIF underscores the need to reexamine current practice patterns and to carefully balance the potential benefits of fixation against the risks of complications, implant-related symptoms, secondary procedures, and healthcare costs. Given the substantial remodeling potential in pediatric and adolescent patients and the often-comparable outcomes reported after operative and nonoperative care, shared decision-making remains essential. Further studies incorporating radiographic characteristics, clinical indications, patient-reported outcomes, complications, and socioeconomic factors are needed to better define appropriate surgical indications and promote more judicious and equitable treatment strategies.

## Figures and Tables

**Table 1:** Annual percentage of ORIF surgeries in the total population and each demographic category.

Year	All Patients	Age		Sex		Race	
		10–14	15–18	Female	Male	Black or African American	White
**2014**	1.62% (137/8457)	0.8% (42/5285)	3.44% (94/2735)	1.20% (22/1829)	2.24% (111/4959)	1.34% (10/746)	2.28% (109/4791)
**2015**	2.35% (209/8909)	1.36% (78/5752)	4.65% (130/2798)	1.58% (32/2019)	3.21% (168/5236)	1.17% (10/855)	3.12% (154/4930)
**2016**	4.45% (256/5752)	2.91% (105/3606)	8.04% (150/1865)	3.55% (39/1098)	5.67% (212/3738)	2.14% (11/514)	6.02% (200/3322)
**2017**	4.21% (281/6682)	2.53% (107/4225)	7.94% (173/2179)	3.72% (48/1291)	5.04% (217/4303)	2.36% (12/508)	5.29% (209/3948)
**2018**	4.72% (311/6586)	3.12% (131/4193)	8.40% (179/2130)	4.45% (58/1304)	5.55% (240/4323)	2.87% (16/558)	5.72% (223/3898)
**2019**	4.60% (328/7130)	3.00% (136/4530)	8.29% (192/2316)	4.25% (60/1412)	5.37% (255/4749)	3.56% (19/549)	5.67% (243/4287)
**2020**	5.62% (308/5476)	3.07% (102/3322)	10.6% (204/1916)	4.17% (48/1152)	6.84% (247/3610)	3.70% (14/378)	6.51% (227/3488)
**2021**	6.11% (457/7474)	3.36% (156/4649)	11.9% (299/2522)	5.44% (81/1490)	6.98% (356/5103)	4.50% (30/666)	7.27% (324/4454)
**2022**	5.63% (428/7598)	2.91% (139/4781)	11.1% (283/2546)	4.68% (68/1454)	4.48% (241/5378)	3.77% (27/716)	6.51% (293/4498)
**2023**	5.83% (442/7578)	3.13% (147/4697)	11.2% (292/2613)	5.74% (81/1412)	6.61% (349/5280)	3.57% (24/673)	6.90% (301/4360)
**2024**	5.89% (389/6609)	2.50% (101/4048)	12.4% (283/2283)	5.83% (76/1304)	6.41% (308/4802)	5.33% (32/600)	7.10% (285/4015)

**Table 2:** Annual Chi-squared (χ^2^) test results assessing the association between demographic categories and surgical intervention type from 2014 to 2024. Comparisons between African American and White groups, Male and Female groups and two age groups: 10–14 years.

Year	African American or Black, White		Male, Female		10–14 YO, 15–18 YO	
	Chi2 (df = 1)	*p*-Value	Chi2 (df = 1)	*p*-Value	Chi2 (df = 1)	*p*-Value
**2014**	2.2	0.133	6.92	**0.008**	73.90	**<0.001**
**2015**	9.4	**0.002**	13.73	**0.0002**	84.46	**<0.001**
**2016**	12.1	**0.0005**	7.32	**0.007**	71.80	**<0.001**
**2017**	7.6	**0.006**	3.57	0.058	99.22	**<0.001**
**2018**	7.3	**0.007**	2.21	0.136	83.31	**<0.001**
**2019**	4.2	**0.040**	2.58	0.107	92.78	**<0.001**
**2020**	4.1	**0.042**	10.30	**0.001**	125.44	**<0.001**
**2021**	6.5	**0.010**	4.17	**0.041**	197.37	**<0.001**
**2022**	7.6	**0.006**	0.06	0.805	204.7	**<0.001**
**2023**	10.2	**0.001**	1.27	0.259	191.1	**<0.001**
**2024**	2.2	0.131	0.50	0.478	249.42	**<0.001**

**Table 3:** Regression Analysis of Annual Percentage Change (APC) in ORIF Procedures.

Demographic Category	APC (%)	*p*-Value	R-Squared
Overall, 1 joint point	5.2	<0.01	0.772
Overall, 2 joint points	−0.8	0.683	0.101
Age 10–14	0.0	0.9823	0.000
Age 15–18	7.5	0.0005	0.843
Female	7.9	0.0007	0.826
Male	2.1	0.3191	0.141
Black or African American	15.0	0.0019	0.768
White	3.4	0.0080	0.658

## Data Availability

The original contributions presented in this study are included in the article. Further inquiries can be directed to the corresponding author.
